# Reduced serpinB9-mediated caspase-1 inhibition can contribute to autoinflammatory disease

**DOI:** 10.18632/oncotarget.8086

**Published:** 2016-03-15

**Authors:** Robert van der Burgh, Jan Meeldijk, Lieneke Jongeneel, Joost Frenkel, Niels Bovenschen, Mariëlle van Gijn, Marianne Boes

**Affiliations:** ^1^ Department of Pediatric Immunology, Wilhelmina Children's Hospital, UMC Utrecht, EA, Utrecht, Netherlands; ^2^ Department of Pathology, University Medical Center Utrecht, CX, Utrecht, Netherlands; ^3^ Department of General Pediatrics, Wilhelmina Children's Hospital, UMC Utrecht, EA, Utrecht, Netherlands; ^4^ Department of Genetics, University Medical Center Utrecht, EA, Utrecht, Netherlands; ^5^ Laboratory of Translational Immunology, University Medical Center Utrecht, CX, Utrecht, Netherlands

**Keywords:** serpinB9, autoinflammation, caspase-1, interleukin 1β, granzyme B, Immunology and Microbiology Section, Immune response, Immunity

## Abstract

Patients who suffer from autoinflammatory disease (AID) exhibit seemingly uncontrolled release of interleukin (IL)-1β. The presence of this inflammatory cytokine triggers immune activation in absence of pathogens and foreign material. The mechanisms that contribute to ‘sterile inflammation’ episodes in AID patients are not fully understood, although for some AIDs underlying genetic causes have been identified. We show that the serine protease inhibitor B9 (serpinB9) regulates IL-1β release in human monocytes. SerpinB9 function is more commonly known for its role in control of granzyme B. SerpinB9 however also serves to restrain IL-1β maturation through caspase-1 inhibition. We here describe an autoinflammatory disease-associated serpinB9 (c.985G>T, A329S) variant, which we discovered in a patient with unknown AID. Using patient cells and serpinB9 overexpressing monocytic cells, we show the A329S variant of serpinB9 exhibits unobstructed granzyme B inhibition, but compromised caspase-1 inhibition. SerpinB9 gene variants might contribute to AID development.

## INTRODUCTION

In patients suffering from autoinflammatory disease (AID), the regulation and release of interleukin (IL) 1β is disturbed. This triggers immune activation in absence of pathogens and foreign material. In AID, these ‘sterile inflammation’ episodes develop in a recurrent manner, characterized by fever and other inflammation-related symptoms. For some AIDs the underlying genetic causes have been identified, however, in many patients the AID cannot be directly related to genetic mutations. On a cellular level, monocytes in AID are primed, leading to an activation-prone status which accelerates and increases the release of IL-1β. The IL-1β processing enzyme caspase-1 cleaves immature IL-1β into its bioactive form, allowing it to perform its pro-inflammatory function [1].

At present, only one endogenous inhibitor of caspase-1 is known, the serine protease inhibitor B9 (serpinB9), which is also the main inhibitor for the cytotoxic effector protein granzyme B (GrB) [2, 3]. In this study we identified a heterozygous A329S variant in the SerpinB9 gene of a patient with AID without genetic diagnosis (periodic high fever (>38°C) with: headache, fatigue and malaise, vomiting, abdominal pain and diarrhea, arthralgia and myalgia in right leg. Patient was treated with paracetamol and diclofenac during episodes. Treatment with colchicine was attempted and failed due to absence of MEFV mutations). We assessed the capability of this serpinB9 protein variant to inhibit both GrB and caspase-1. Whereas the serpinB9 mutant fully retained its ability to block GrB, it failed to fully inhibit caspase-1 activity. These data provide the first functional evidence that mutations in serpinB9 can contribute to AID, while retaining full capacity at inhibition of GrB.

## RESULTS AND DISCUSSION

**Figure 1:**
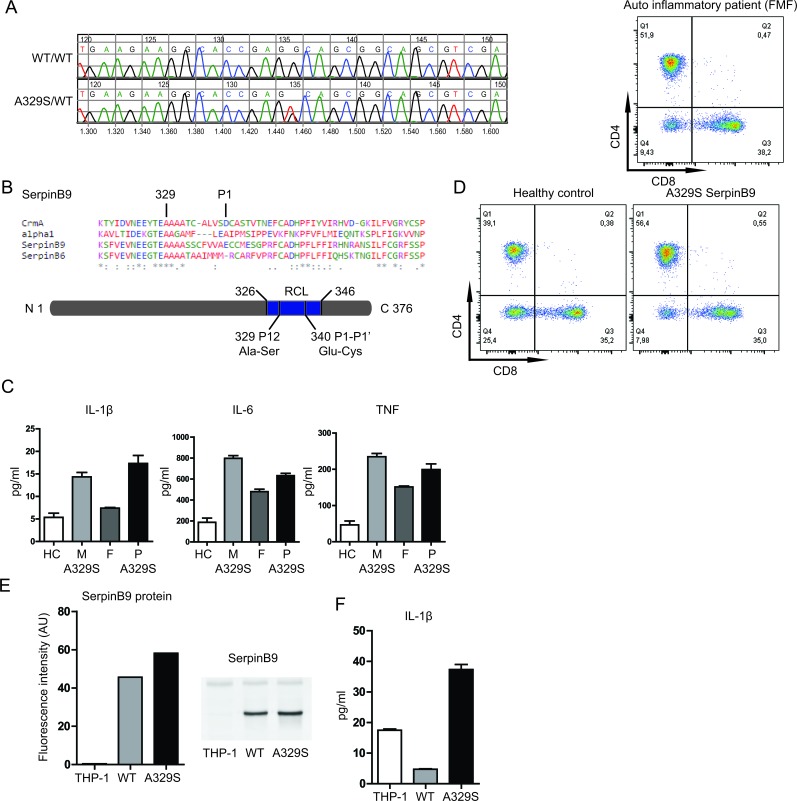
Effects of serpinB9 mutation in patients and THP-1 cell line **A.**, Results for Sanger sequencing showing the mutation in serpinb9. **B.**, Alignment of four members covering the serpin superfamily, with the conserved residue (329) and cleavage site (P1) of serpinb9 highlighted. **C.**, IL-1β, IL-6 and TNF secretion of LPS stimulated PBMCs from patient and family. **D.**, CD8 T-cell counts of patient and healthy control. **E.**, SerpinB9 protein levels by fluorescence intensity and Western blot (representative of two independent experiments) **F.** IL-1β secretion by serpinB9 overexpressing and control THP-1 cells (after stimulation with 200 ng/mL LPS for 4 hrs; four independent experiments, three biological replicates).

We performed a genetic screening of autoinflammatory patients of unknown origin for disease-associated gene variants, using a targeted Next-Generation Sequencing-based approach [4]. Here, we report a Serine to Alanine replacement at position 329, in SERPINB9 (NM 004155.4, c.985G>T, Figure 1A). The mutation is located in a highly conserved region of the serpin superfamily (Figure 1B). The mutation is not present in control populations from European ancestry but has a minor allele frequency of 0.006 in the Latino population [5]. Analysis of the parents showed that the mutation was inherited from the mother. The mother indeed self-reported suffering from AID symptoms in her youth particularly. Spontaneous resolution or reduction of AID symptoms during adolescence is not uncommon and might explain the current lack of symptoms for the mother.

We asked whether a disease-associated gene variant in serpinB9 might contribute to AID pathology. To this end, we first evaluated the stimulation-induced cytokine release from peripheral blood mononuclear cells (PBMCs) of the patient and parents. Stimulation (with 200 ng/ml lipopolysaccharide (LPS), 4 hours) resulted in increased IL-1β, IL-6 and TNF secretion by the PBMC harboring the A329S serpinB9 mutation (Figure 1C; M, mother and P, patient), compared to a family member lacking the mutation (F, father).

We considered that defects in serpinB9 function can be associated with decreased numbers of circulating CD8 T-cells, as a consequence to increased sensitivity to apoptosis [6]. We therefore next evaluated the relative abundance of CD8 T-cells in the peripheral blood. These cells contain significant amounts of GrB and if GrB inhibition is negatively affected, there will be an expected decrease in the CD8 T-cell numbers [3]. Yet the percentage of CD8 T-cells (35.0%) is almost identical to an unrelated healthy control and well within normal range. As additional control sample, we evaluated an unrelated AID patient with Familial Mediterranean Fever (FMF) using the same assay. Also the FMF patient showed a comparable percentage of CD8 T-cells (38.2%), supporting that neither defects in GrB inhibition nor the presence of AID results in decrease in the presence of CD8 T-cells in the circulation (Figure 1D).

To further investigate the properties of the serpinB9 mutant in isolation and without possible genetic background-associated confounding factors, we produced two monocytic THP-1 cell lines overexpressing both the healthy and AID-associated serpinB9 gene variants. We first quantified the serpinB9 protein levels in these monocytic lineage cells (Figure 1E), which confirmed overexpression of serpinB9 when compared to regular THP-1 cells, with the mutant expression being approximately 1.3 times higher than the wild type. We stimulated the cells (LPS 200 ng/mL, 4 hr) and assessed if the cells were still able to secrete IL-1β (Figure 1F). As expected, overexpression of wild type serpinB9 yielded much reduced IL-1β when compared to the non-transfected cells. The AID-associated serpinB9 variant was defective at inhibition of IL-1β production, even causing an increase in secreted IL-1β. These data support that the A329S variant of serpinB9 associates with IL-1β hypersecretion by monocytic cells.

**Figure 2:**
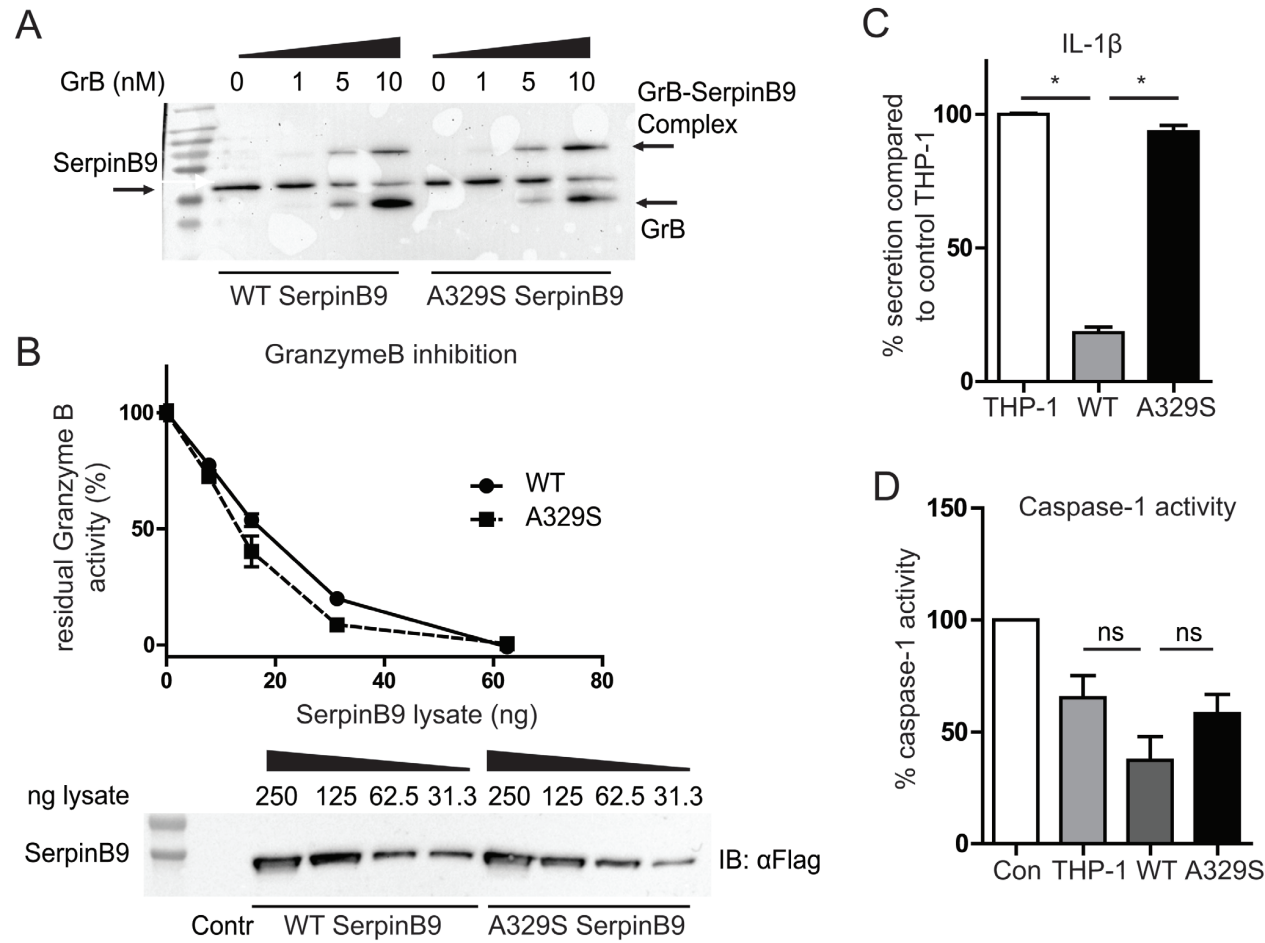
Inhibition of GrB and Caspase-1 by wild type and A329S serpinB9 **A.**, SDS-resistant complex of GrB and serpinB9 is formed by both variants. Representative of three experiments. **B.**, Kinetic measurement of GrB inactivation. The kinetics are comparable between wild type and mutant. Representative of three experiments. **C.**, IL-1β secretion for serpinB9 overexpressing, simvastatin treated THP-1. The wild type but not the mutant serpinB9 effectively reduces IL-1β secretion by THP-1 cells. Graph is the average of three independent experiments. **D.**, Caspase-1 activity measure with THP-1 lysate and recombinant caspase-1. The mutant serpinB9 retains more caspase-1 activation compared to the wild type, while protein levels are similar. Average of four independent experiments. * = *p* < 0.05 (Wilcoxon matched-pairs signed rank test)

The reactive center loop (RCL) of serpins has been intensely studied and most mutations in this loop have detrimental effects on the inhibition of the target [7, 8]. Considering that the mutated alanine is in the RCL and highly conserved, we expected a difference in serpinB9 binding to GrB. Such an effect would likely disrupt the serpinB9 ability to block GrB, and might reveal a similarity in mechanism to inhibit Caspase-I. To address this possibility, we took a biochemical approach, in which we introduced increasing amounts of recombinant GrB into serpinB9 containing THP-1 lysates. Western blot shows protein complex formation of GrB with both the wild type and the mutant forms of serpinB9, indicating there was no defect in binding of the mutant serpinB9 to GrB. To determine the kinetics of the inhibition, we measured GrB activity in 293F lysates with different concentrations of serpinB9. GrB was added to the lysates and the residual activity of GrB was assessed (Figure 2B). Again, there was no significant difference, indicating that, despite the RCL mutation, both serpinB9 variants inhibit GrB activity and their inhibition proceeds with similar kinetics.

We next asked whether the inhibition of caspase-1 was affected by the AID-associated serpinB9 variant. We therefore wished to measure IL-1β secretion as readout for caspase-1 inhibition, considering that caspase-1 is a requirement for mature IL-1β to be secreted by cells. THP-1 cells, however, only secrete modest amounts of IL-1β in response to LPS stimulation. THP-1 cells that overexpress SerpinB9, and thus inhibit caspase-1 enzymatic function might express even less IL-1β, precluding any possibility to functionally gauge caspase-1 function. To address this issue, we resorted to the use of simvastatin, a common cholesterol-lowering drug, that when added together with LPS strongly potentiates IL-1β secretion by monocytic cells. Indeed, in earlier AID research using human monocytes and THP-1 cells, we and others have successfully used simvastatin to boost caspase-1-dependent IL-1β secretion [9, 10]. We therefore pretreated the cells with 10μM simvastatin (24 hours), followed by stimulation with LPS (200 ng/mL, 4 hr). Control THP-1 cells responded with a greatly increased IL-1β secretion. In contrast, the THP-1 cells overexpressing wild type serpinB9, showed a clear functional inhibition, with a restrained increase in secreted IL-1β ranging between 5 pg/mL and 40 pg/mL (Figure 2C). The increased IL-1β production by THP-1 cells overexpressing the A329S serpinB9 showed levels that were comparable with the levels seen in control THP-1, supporting defective inhibition of caspase-1 (Figure 2C).

Finally, to directly investigate if the mutant form of serpinB9 is less proficient at inhibiting caspase-1 we spiked the lysates of serpinB9-overexpressing THP-1 cells with recombinant human caspase-1. We assayed the remaining caspase-1 activity in a colorimetric assay. The lysate of control THP-1 cells contained some intrinsic inhibitory effect on caspase-1 activity, compared to the control (no lysate). Lysate with mutant serpinB9 showed a similar rest activity for caspase-1. In contrast, the wild type serpinB9 variant lysate showed lower rest activity, supporting more functional inhibition capacity of caspase-1 by the wild type serpinB9 compared to the AID-associated serpinB9 (Figure 2D). We conclude that the A329S serpinB9 mutant has decreased proficiency at inhibiting caspase-1, while its inhibitory capacity of GrB is unaltered.

Taken together, we here describe a novel AID-associated protein, SerpinB9. In a monocytic model cell system we overexpressed and evaluated the function of the mutant serpinB9 protein. GrB binding and inhibition was equivalent to the wild type serpinB9. However the ability to control caspase-1 activity was diminished for the AID-associated serpinB9 gene variant when compared to the wild type serpinB9. We show this in functional assays in which we measured IL-1β secretion as well as by measuring rest activity of Caspase-I directly in lysates expressing wild type or mutant SerpinB9. We conclude that the serpinB9 function controls Caspase-I function and when mutated, might contribute to the pathogenesis of AID.

## MATERIALS AND METHODS

### Reagents

Simvastatin was purchased from SIGMA-Aldrich. Caspase-1 colorimetric assay kit and recombinant human caspase-1 were purchased from Biovision. LPS (Escherichia coli EH 100) was obtained from Alexis Biochemical. Simvastatin was hydrolyzed to its bioactive form as previously described [11].

### Antibodies

FACS: CD3-APC, CD8-PerCP-Cy5 (BD biosciences, 555335 & 341050) CD4-PB (Biolegend, 300521). Western blot: Actin (Santa Cruz, sc-1616). Anti-FLAG (clone M2, Sigma Aldrich, F3165). Secondary antibodies for Odyssey: IRDye800 Donkey-anti-mouse and IRDye680 (Li-Cor, 925-32212 and 925-68073). Recombinant GrB was expressed and purified as we described previously [12, 13] and serpinB9 antibody (clone PI9-17) was made as described [14].

### Patient samples

Patient was a 6 year old male with AID, recurrent episodes of high fever and generalized inflammation as reflected by elevated acute phase proteins in the absence of infection. He was therefore diagnosed with autoinflammatory disease. However, in none of the genes known to cause AID could we identify mutations. Parents were non-consanguineous and reportedly healthy. Although the mother did experience recurrent febrile illnesses as a child, it is unknown whether or not these had been related to infections. At scheduled outpatient visits, the patient was afebrile and well, underwent routine blood analysis. The ethical committee of the UMC Utrecht approved the use of residual material for this study. Residual material from routine blood tests was used to obtain peripheral blood mononuclear cells (PBMC). PBMC from patient, parents, and healthy donors were isolated using ficoll density gradient. PBMC fraction was washed twice in RPMI supplemented with 2% FBS and used immediately.

### Sequencing of 120 inflammasome related genes

Barcoded whole genome fragment library were generated, and enriched for the coding regions of 120 inflammasome genes using a custom Agilent 1M microarray. The enriched library was sequenced on the SOLiD4 sequencer as described previously [4, 15]. The detected SerpinB9 variant was confirmed with Sanger sequencing. Primer sequences are available upon request.

### Cell cultures

Freestyle 293F cells (Life Technologies) were cultured according to manufacturer's protocols. THP-1 cells were cultured in RPMI 1640 supplemented with 1% glutamine, antibiotics (penicillin, streptomycin) and 10% FBS. Simvastatin treatment of cells was 24 hours prior to the start of the experiment and at a concentration of 10 μM.

### Cloning and mutagenesis

The mutant form of SerpinB9 was generated by site-specific mutagenesis of the wild type SerpinB9 (Source Bioscience, IRAUP969C028D). First, DNA from SerpinB9 was amplified for further cloning using primer serpinB9 fw and serpinB9 rev. The amplicon was ligated into the pGEM-T vector (Promega) and sequenced. The mutation was entered with a PCR reaction according to the guidelines of Phusion DNA polymerase (Finnzymes, F-5302S) adding 2 U/ 50ul reaction Phusion, 100ng template DNA and primer serpinB9 A329S fw and serpinB9 A329S rev. Template (methylated) DNA was degraded by adding 1 μL DpnI (NEB, R0176s) and incubated overnight at 37°C. Wild type and mutant vector were transformed in commercial DH5a (NEB, C2987I) and the mutations were confirmed by sequencing using primer serpinB9 seq. Both the wild type serpinB9 and A329S serpinB9 variant were cloned with XhoI and NotI (NEB, R0146L and R0189L) into the retroviral vector pMSCV-puro from which the multiple cloning site was altered. The final constructs were confirmed with Sanger sequencing. Used primers: serpinB9 fw 5′- CAC ACT CGA GGC CGC CAC CAT GGA AAC TCT TTC TAA TGC AAG - 3′, SerpinB9 rv 5′ - CAC AGC GGC CGC TTA TGG CGA TGA GAA CCT G - 3′, serpinB9 A329S fw 5′ - GAA GGC ACC GAG TCA GCG GCA GC - 3′, SerpinB9 A329S rv 5′ - GCT GCC GCT GAC TCG GTG CCT TC - 3′, SerpinB9 seq 5′ - GAG AGA GAC CTG TGT CT - 3′

### Transduction and selection of stable THP-1 serpinB9 lines

Wells of a 6 wells plate were coated for 2 hours at RT with 2 ml 50 μg/ml Retronectin (Takara Bio inc., T100B). Retronectin was removed and the plates were blocked with 2 ml 2% BSA/ PBS for 30 minutes at RT, followed by 2 washing steps with PBS. The retroviral constructs were transfected in Phoenix-ampho cells, producing the retrovirus. The retrovirus-containing sup was loaded on Retronectin-coated plates and centrifuged for 1,5 hour at 1800x g, 4oC. The supernatant was removed and 8*105 THP-1 cells were added with the addition of 8 μg/ml polybrene. The plate was briefly centrifuged and thereafter incubated overnight at 37oC. The day after the infection with retrovirus was repeated on the same cells. Overexpressing cells were selected with 0.5 μg/ml puromycin.

### Flow cytometry

Isolated PBMCs were spinned and resuspended in FACS buffer and stained for CD3, CD4, CD8 and CD14. Cells were kept in the dark until measurement on FACS CANTO-II. Analysis was done with FACS Diva software.

### Cytokine measurements

Cells were centrifuged (500g, 5 min) and plated in 96 well plates in triplicates (2.0 * 105 cells/well in 200 μL), followed by 1 hour incubation at 37°C. Next LPS (200 ng/mL) was added and supernatants were collected after 4 hours and stored at −80°C until measurement. Cytokine concentrations were determined by Mulitplex bead analysis and normalized to the percentage of monocytes in the blood.

### Immunoblot analysis

Cells were washed twice in PBS and then resuspended in laemmli buffer and boiled for 10 min. Samples were then distributed in small aliquots and stored at −20°C until use. Protein content was determined with BCA assay and samples diluted to 1μg/μL. 5% v/v β-mercaptoethanol was added to the samples and they were separated on 12% SDS PAGE gel, followed by transfer to PVDF-FL membrane. 5% dried non-fat milk was used for blocking followed by primary antibody incubation (overnight 4°C, 0.5% milk in TBS-T), three washes and secondary antibody incubation (1 hr RT, 0.5% milk in TBS-T). Detection was done with enhanced chemiluminescence (ECL) on film or with Biorad Chemidoc MP. Some blots were visualized with labeled secondary antibodies and measured with Odyssey scanner.

### Caspase-1 activity measurements

We used the colorimetric caspase-1 activity detection kit (biovision). THP-1 cells and THP-1 lines overexpressing serpinB9 wild type and mutant were lysed according to manufacturer's instructions. Protein content was determined with BCA assay of THP-1 lysate and THP-1 wild type serpinB9 lysate per well was used. The amount of mutant serpinB9 THP-1 lysate was adapted to get equal serpinB9 amounts based on Western blot protein amount determination. Assay was performed according to manufactures instructions with one addition. Recombinant human caspase-1 (Biovision, 1081-25) was added to the lysate (0.5 unit per well). Measurement was done with plate reader using a 405nm filter.

### GranzymeB inhibition

Freestyle 293F cells were transfected according to manufacturer's protocol with either pBicDNA-FlagHA-SerpinB9 or pBicDNA-FlagHA-SerpinB9-A329S. Lysates were prepared 3 days post transfection. Cells were washed 3 times in cold PBS (5 min, 300g, 4°C) followed by 3 Freeze/Thaw cycles in PBS using liquid Nitrogen. Lysates were cleared by centrifugation (10000g, 10 min, 4°C) and stored at −20C. Protein concentration was determined by Bradford method. Lysate was pre-incubated with GrB for 2 hours at 37°C under mild shaking (BMGlabtech Thermostar, 150 rpm) in a volume of 50 μl per well (96 well plate). Different concentrations of SerpinB9 containing lysate were used; total lysate concentration was kept at 250 μg/ml by mixing with control 293F lysate. The GrB substrate (Ac-IEPD-pNA, ENZO Lifesciences, BML-P133-0005) was added to the wells in 10 μl solution (1.25 mM substrate, 100 mM Tris-Cl pH7.4, 200 mM NaCl, 0.01% Tween20). Final GrB concentration was 20 nM. Substrate conversion was measured by optical density at 405 nm with Anthos Zenith 340 rt plate reader at 37C in kinetic mode and the initial slope was determined.
